# Wild Food and Medicinal Plant Knowledge Amidst Biocultural and Linguistic Rarefaction: A Diachronic Ethnobotanical Study of Walser-Speaking Alpine Communities of NW Italy

**DOI:** 10.3390/foods15213994

**Published:** 2026-11-07

**Authors:** Corinna Debiaggi, Hudrah Bagaliza, Gaia Cappelletti, Zita Sartori, Giulia Mattalia, Naji Sulaiman, Andrea Pieroni

**Affiliations:** 1 University of Gastronomic Sciences of Pollenzo, Piazza Vittorio Emanuele 9, 12042 Bra, Italyg.cappelletti@studenti.unisg.it (G.C.);; 2 Sartori AgriBioCultural Solutions, 6685 Bosco/Gurin, Ticino, Switzerland; 3 Laboratori de Botànica–Unitat Associada CSIC, Facultat de Farmàcia i Ciències de l’Alimentació–Institut de Recerca de la Biodiversitat (IRBio), Universitat de Barcelona, 08028 Barcelona, Spain

**Keywords:** local ecological knowledge, food heritage, ethnobotany, Alps, minority language, wild food plants, socio-ecological resilience, biocultural diversity

## Abstract

The Alps, the highest mountain range of Europe, shared by seven countries, are characterized by high biocultural diversity fostered by their geography. Among the several distinct groups inhabiting them, the Walser are Alemannic communities who settled in the Western Alps during medieval times and are recognized for their distinctive architecture, land management, and German-rooted languages. We present here a diachronic ethnobotanical study of Walser communities in the Italian Alps, documenting 43 plant taxa traditionally used as food and medicine. Drawing on semi-structured interviews, focus groups, and participant observation, we trace the accelerating erosion of traditional wild-food knowledge and the food practices associated with it along three converging axes: demographic depopulation, language attrition, and the collapse of subsistence food systems and related land management. The ethnobotanical comparison reveals a rapid narrowing of the knowledge horizon: active wild-plant knowledge holders are now generally over 60, core culinary species are retained in cultural memory but increasingly less used, and local varieties of the Walser language, which encode ecological and nutritional distinctions with no direct Italian equivalents, are critically endangered. These processes threaten not only biocultural heritage but also the continuity of locally embedded food practices and intergenerational knowledge transmission. We argue that the revitalization of traditional wild-plant food practices may provide a socially sustainable entry point for strengthening community resilience, reconnecting generations with local environments, and safeguarding broader bodies of at-risk biocultural knowledge.

## 1. Introduction

The Alps are not a wilderness area: they are a cultural landscape, shaped over centuries by communities who learned through intimate daily contact with mountain environments in which plants could sustain a household through a long Alpine winter, temper a fever, or mark the arrival of spring [1,2]. In Alpine communities, traditional and local ecological knowledge (LEK), the cumulative body of knowledge, practices, and beliefs about relationships among living beings and their environment, evolved through adaptive processes and transmitted through cultural learning across generations, is not a static archive [3] and has been critical for survival. Rich LEK sustains ecosystem resilience by maintaining the biodiversity and habitat heterogeneity on which adaptive capacity depends [3,4]. LEK is crucial for distinguishing edible from toxic plants, knowing which herb provides an expectorant in midwinter, or managing communal pastures to prevent overuse [5,6].

Among the most remarkable of the communities who shaped the Alpine landscapes are the Walser, Germanic-speaking groups who migrated southward from the Upper Valais (Switzerland) beginning in the thirteenth century and established settlements across some of the harshest terrain in the (Italian) Alps [1,7]. Their success depended on an integrated suite of adaptive practices, distinctive timber architecture, communal land governance, finely calibrated pastoralism, and an encyclopaedic knowledge of local flora, accumulated and transmitted across centuries of mountain life. Today, the Walser communities, which still inhabit the Italian, Swiss, Liechtenstein, and Austrian Alps, occupy a paradoxical position. Their traditional architecture features in tourist brochures; fragments of their language appear in regional identity politics [8]; their festivals attract second-home owners and cultural tourists [8,9,10]. But the continuity of this living heritage is increasingly vulnerable to demographic, linguistic, socioeconomic, and ecological change [11,12].

A defining feature of Walser LEK is its deep linguistic encoding. The Walser language, differentiated into mutually distinct local varieties, reflecting centuries of geographic isolation, contains botanical and culinary vocabulary with no equivalent in Italian or standard German. Terralingua’s concept of biocultural diversity captures this entanglement: biological and cultural diversity co-evolve and mutually sustain each other [13]. Hunn [14] describes this as ‘ecological indexicality’, the capacity of a language to point precisely towards entities and relationships in the natural world. Pieroni and Giusti [15], working with Occitan communities in the Piedmont Alps, demonstrated that linguistic diversity directly supports the vocabulary needed for precise botanical transmission. Recent ethnobotanical scholarship increasingly suggests that wild food knowledge should not be understood merely as a static body of transmitted information, but as a dynamic socio-ecological practice shaped by ecological availability, cultural exchange, mobility, and changing food systems [16]. Diachronic studies in Alpine and Mediterranean regions have shown that foraging repertoires may persist, contract, or adapt depending on whether daily embodied interaction with landscapes remains active [17]. In the Western Alps, longitudinal comparison demonstrated that core culturally salient taxa often persist even as peripheral taxa disappear under socioeconomic transformation [17]. Similarly, recent Mediterranean studies have highlighted that plant knowledge may persist linguistically or culturally even after active gathering declines, resulting in situations in which botanical labels remain in collective memory while practical knowledge becomes fragmented [16,18]. These findings suggest that the erosion of ethnobotanical heritage is not solely a matter of species loss, but also of weakening ecological practice.

Recent digital resources further demonstrate the growing importance of systematic documentation of wild edible plants in Italy. The AlimurgITA database provides an accessible resource for researching the Italian alimurgic flora and brings together information relevant to the identification and study of wild edible plants. Such resources are particularly valuable for contextualizing local ethnobotanical knowledge within the broader Italian flora and for supporting comparisons among regions and communities. In the present study, the focus is narrower, concentrating on Walser-speaking communities and the relationship between wild-plant uses, local phytonyms, and the continuity and transformation of ethnobotanical knowledge [19].

Although these studies provide evidence of transformation in ethnobotanical practices, assessing the magnitude and rate of local ecological knowledge (LEK) erosion requires research designs capable of comparing knowledge across generations or through repeated sampling of the same communities. The present study was not designed as a systematic intergenerational assessment. Instead, it combines contemporary ethnobotanical documentation with previously collected information to examine patterns of continuity and change in plant knowledge, uses, and local phytonyms. LEK erosion is therefore considered here as a broader contextual issue and an interpretive framework rather than as a directly quantified outcome.

Therefore, the research objectives of this study were: (1) to document local ecological knowledge linked to wild food and medicinal plants across three Walser communities; (2) to identify commonalities and divergences in Walser ethnobotanical knowledge across communities and in comparison with previously published Walser data, including local phytonyms; and (3) to explore patterns of continuity and change in Walser ethnobotanical knowledge through comparison with previously published and historical information. Moreover, we discuss the ethnobotanical findings within the broader literature on biocultural resilience and consider whether traditional food heritage may provide a context for maintaining and transmitting local ecological knowledge.

## 2. Materials and Methods

### 2.1. Study Sites and Historical Context

The Walser originated in the Upper Valais region of present-day Switzerland and began migrating southward in the early thirteenth century, establishing colonies across the most challenging terrain of the Western and central Alps [7]. In Valsesia alone (Piedmont, Italy), they founded seven main colonies: Alagna, Rima, Rimella, Riva Valdobbia, Rimasco, Carcoforo, and Fobello [20]. Their adaptive practices included stone terracing, distinctive three-story timber architecture integrating stables, living quarters, and hay storage for thermal efficiency, and the management of communal pastures and resources through formally organized consortia [21]. The Walser language (wae, its ISO 639-3 code [22]) is highly differentiated across communities into mutually distinct local varieties, as they evolved in relative geographic isolation over approximately eight centuries, surviving today mainly among some elderly community members [23,24].

### 2.2. Fieldwork Sites and Methods

**Figure 1:**
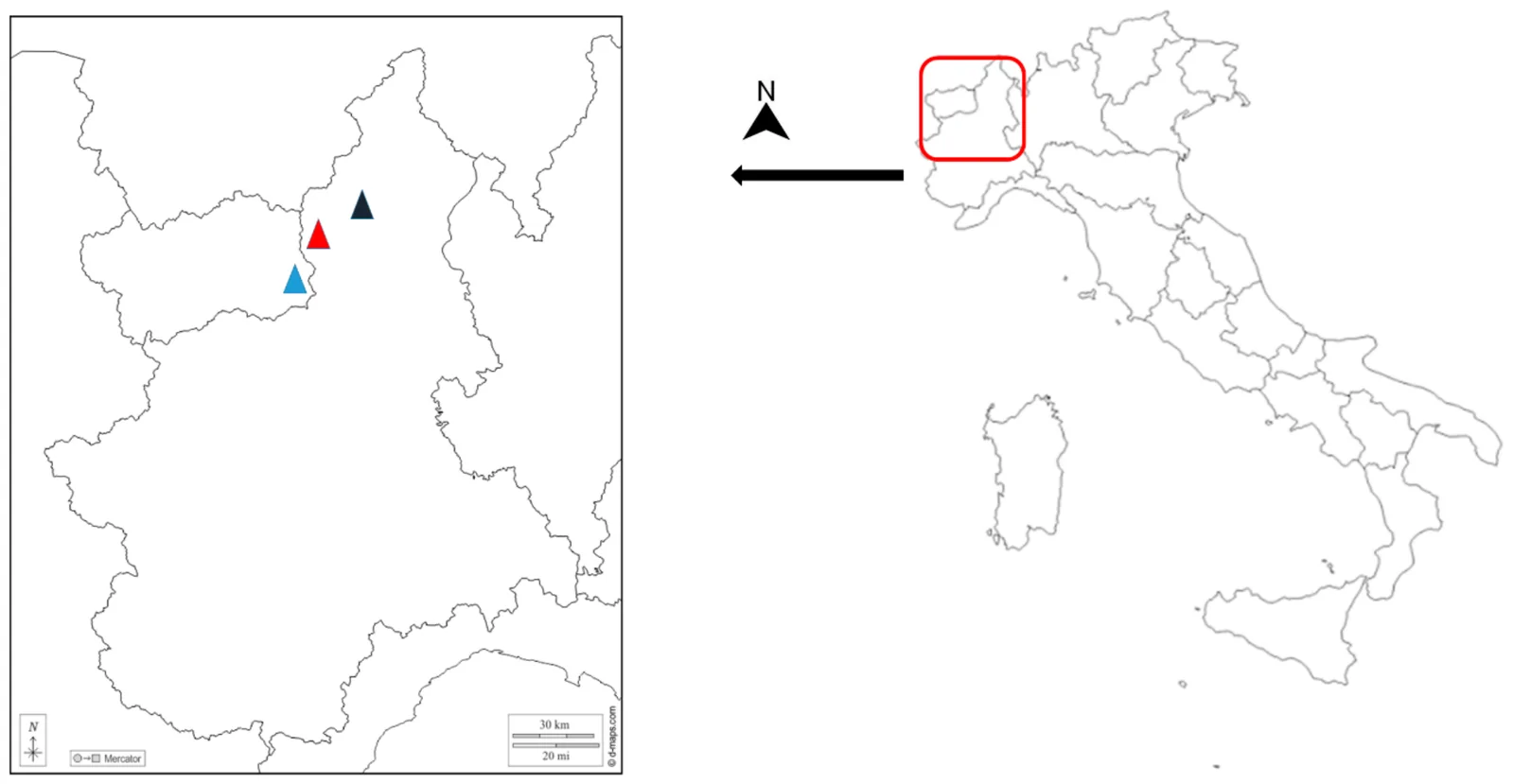
Map of the three Walser-speaking communities included in the ethnobotanical inventories: Issime in the Aosta Valley and Alagna and Rimella in Valsesia, Piedmont. Supplementary contextual ethnographic research was also conducted in Val d’Otro in 2016.

Ethnobotanical inventories were compiled for three Walser-speaking communities: Issime in 2010 and Alagna and Rimella in 2026. Additional contextual ethnographic research was conducted in Val d’Otro in 2016 (Figure 1). Because the Val d’Otro interviews focused primarily on socioeconomic change rather than comprehensive plant documentation, this material is treated as supplementary contextual evidence rather than as a fourth ethnobotanical inventory. The main topic of the semi-structured interviews was foraging and its transformation and the detailed foraged wild plants for food and herbal scopes.

Issime–Éischeme, Aosta Valley. Issime (ca. 400 inhabitants; ca. 1000 m a.s.l.) lies in the Lys Valley. Its local Walser variety, Töitschu, differs substantially from the Valsesian varieties, reflecting centuries of separate evolution. Fieldwork, conducted in 2010, used informal interviews with 15 elderly women, supplemented by plant-collection walks at the Vallone di San Grato and the Vallone di Montil. Sessions of free-listing and participant observation were designed to be as naturalistic as possible, without questionnaires or recording equipment, allowing associative recall through conversation. A painting of wild plants used in local cooking, assembled by an elder, served as a key discussion prompt. Sessions were conducted in Italian.

Alagna Valsesia–Im Land, Valsesia, Piedmont. Alagna (700 inhabitants; ca. 1200 m a.s.l.) is the principal inhabited Walser settlement in Val Grande, probably founded around 1285 [25,26]. Here we gathered data in 2026 through a focus group of six participants aged 69–88, facilitated by a local Walser linguist, using a semi-structured format moving from culinary to medicinal and ritual plant uses. All sessions were recorded with consent; detailed field notes were taken.

Rimella–Remmalju, Valsesia, Piedmont. Founded in 1256, Rimella (140 inhabitants, ca. 1200 m a.s.l.) is the oldest Walser settlement in Valsesia and the most severely depopulated: there were approximately 1000 inhabitants in the sixteenth century [20]. Data were collected in 2026 through 16 individual interviews facilitated by the local museum curator, who also conducted approximately ten brief interviews with additional inhabitants.

In addition, in 2016, we conducted two multi-day field visits and eight semi-structured interviews in Val d’Otro–Oltertol. Val d’Otro is a locality in Valsesia, Piedmont, with no permanent inhabitants, lying at approx. 1700 m a.s.l., accessible only via a one-hour hike from Alagna. The interviews were conducted in Italian and partially in English; all were audio-recorded with consent. In this study, data from the Val d’Otro document the socioeconomic drivers of LEK loss rather than producing a comprehensive plant inventory. The datasets therefore differ in sampling design, participant composition, interview format, recording procedures, and research objectives. In particular, the 2010 Issime fieldwork, the 2016 Val d’Otro research, and the 2026 Alagna and Rimella fieldwork were not designed as repeated surveys of the same population. The historical and contemporary datasets are consequently used for qualitative comparison rather than as directly equivalent measurements of temporal change. Their comparison is intended to identify patterns of continuity, divergence, and reported transformation in ethnobotanical knowledge (Table 1).

### 2.3. Data Analysis and Nomenclature

A unified plant database was compiled, listing scientific names, botanical families, Walser names across all three varieties, parts used, and local use. Plant identification was facilitated by consulting the voucher specimens deposited at the University of Gastronomic Sciences during a previous ethnobotanical study conducted in a surrounding area [15,27]. Current scientific nomenclature was verified against POWO (Plants of the World Online) [28]; families follow APG IV (2016). Walser names were verified with the support of a local language expert and cross-referenced with the FloraWalser database [29]. Participants were informed of the purpose of the research and gave informed consent for recording where needed and for the use of their contributions in research publications. Quotations are presented in accordance with the consent provided and the agreed confidentiality arrangements. Throughout the manuscript, the term “taxon/taxa” is used as an umbrella term for the entries in the compiled inventory. The entries are not all resolved to the species level: the inventory includes a subspecies, species aggregates, and a combined identification for which species-level resolution was not possible. These differences in taxonomic resolution are retained and indicated in the inventory.

We compared the list of wild medicinal species recorded in Rimella and Alagna with a previously published document. The ethnographic monograph of Remogna [30], compiled from interviews with elderly Rimellese informants in the early 1990s, represents the earliest systematic record of folk medicine and everyday food at Rimella and documents practices reaching back to the mid-twentieth century. We also calculated the Jaccard Index according to González-Tejero et al. [31].

The Jaccard similarity index was used to assess the compositional overlap between the medicinal-plant genera recorded in the contemporary Alagna and Rimella dataset and those reported in the historical inventory by Remogna [30]. Each genus was coded as present or absent in the respective inventory. The index was calculated as (J = a/(a + b + c)\), where \(a\) represents the number of genera shared by both inventories and \(b\) and \(c\) represent genera occurring in only one inventory. Because the historical and contemporary datasets differ in scope and collection methods, the index is interpreted as a descriptive measure of compositional similarity rather than a direct measure of temporal knowledge loss.

## 3. Results

### 3.1. Overview of the Ethnobotanical Findings

Across the three ethnobotanical study communities, we documented 43 plant taxa with culinary and/or medicinal uses, belonging to 26 botanical families. Of these, 29 taxa were reported for food use and 18 for medicinal use, with four taxa represented in both categories. The most represented family is Asteraceae (12 taxa), followed by Polygonaceae (4), Apiaceae (3), Caryophyllaceae (3), and Ranunculaceae (3). Culinary uses constitute the largest category (F; 28 taxa), followed by medicinal (M; 20 taxa); five taxa are assigned to both categories. Table 2 presents the complete alphabetically ordered dataset.

### 3.2. The Wild Spring Pantry: Food Uses, a Comparison Across the Studied Communities

The culinary dimension of Walser wild-plant knowledge is inseparable from the community’s historical relationship with seasonality and the nutritional rhythm of Alpine life. Wild plants were collected and consumed almost exclusively in spring, the brief window between the end of a winter diet based on potatoes, milk, cheese, and dried meats and the availability of garden produce. In the words of one Alagna participant, spring foraging was *‘un modo per ripulirsi dall’inverno’*, a way to cleanse the body after winter, a phrase capturing both the physiological reality of a diet suddenly rich in vitamins and minerals and the ritual significance attached to the seasonal return of the landscape. This framing was consistent with accounts from Issime in 2010, where elderly women described the same seasonal transition in almost identical terms.

The central culinary preparation across all communities is a mixed-herb soup assembled from whatever young spring leaves were available. The species most consistently mentioned across all sites and all three fieldwork periods were *Blitum bonus-henricus* (Haimini [A]/Malpene [R]/Lucche [I]), *Silene vulgaris* (Spïchal [A]/Schpeckene [R]/Kwicki [I]), *Urtica dioica* (Nessia [A]/Nassju [R]/Néssju [I]), and *Rumex alpinus* (Blacka [A]/Blàkchtu [R]/Blackhu [I]). In Alagna, this preparation took a locally distinctive form: the *achmil bangada*, a soup made not with water or stock but with the acidic buttermilk (*achmil*) remaining after butter production, which must not contain carrots or legumes. A particularly revealing feature of Walser culinary plant classification is the distinction between ‘lean’ vegetables (*verdure magre*) and ‘fat’ vegetables (*verdure grasse*). *Phyteuma spicatum* (Zunge [A]), which turns black when cooked due to polyphenol oxidation, was classified as lean and nutritionally insufficient alone; *Silene vulgaris* was classified as fat and considered a complete dish.

**Figure 2:**
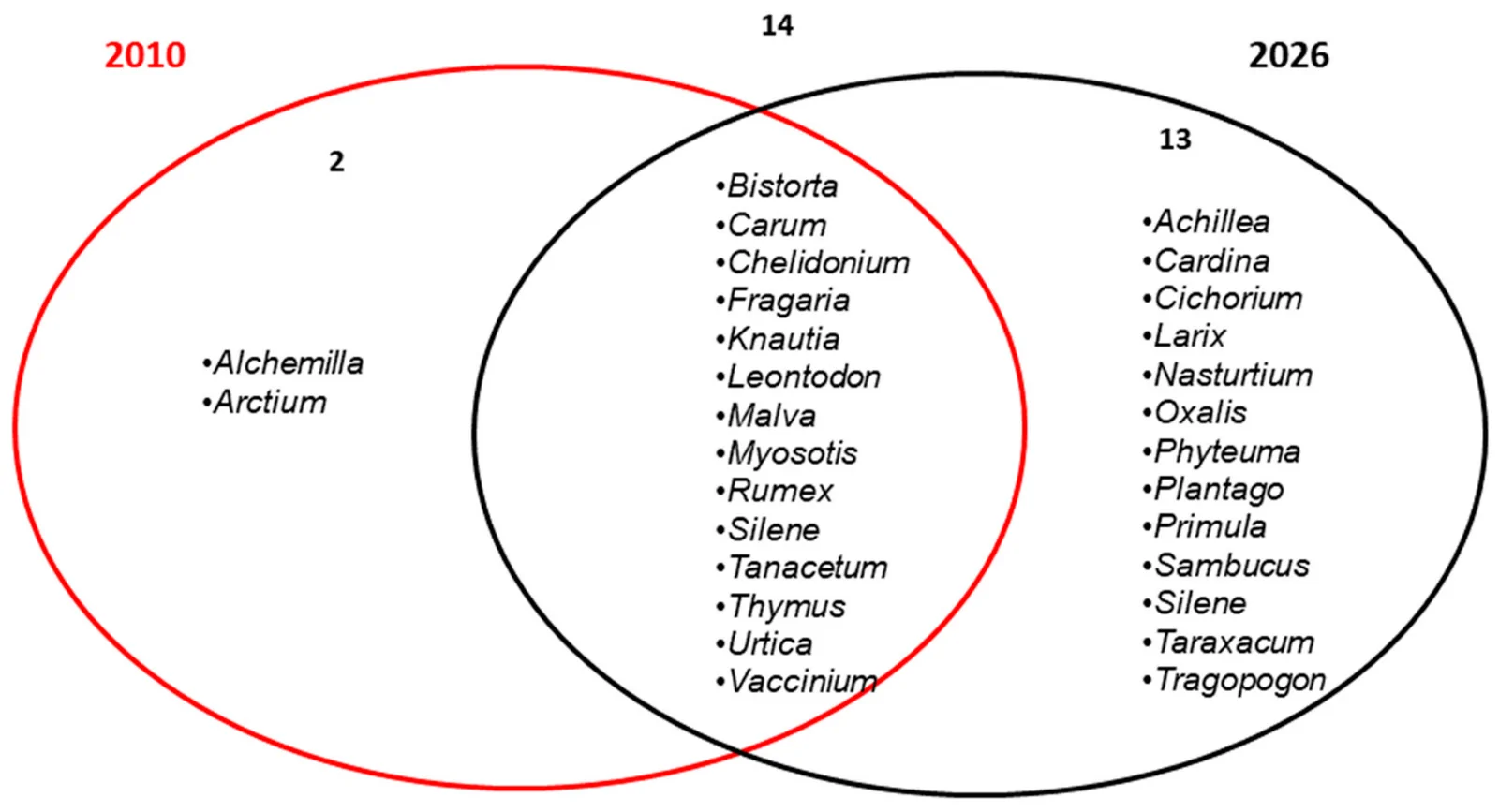
Venn diagram of wild food plant genera documented at Issime (2010, n = 16 genera) and Alagna and Rimella (2026, n = 27 genera). Wild food (culinary) uses only; medicinal-only genera are excluded from both datasets.

The genus-level Venn comparison of wild food plants across the two valleys (wild food uses only; n = 16 genera at Issime, Aosta Valley 2010, n = 27 at Alagna and Rimella, Valsesia 2026) reveals that fourteen genera form the stable shared core (*Bistorta*, *Carum*, *Blitum*, *Fragaria*, *Knautia*, *Leontodon*, *Malva*, *Myosotis*, *Rumex*, *Silene*, *Tanacetum*, *Thymus*, *Urtica*, and *Vaccinium*) documented as wild food across all sites and both time points (Figure 2).

Two genera are present in the 2010 Issime dataset but absent from the 2026 Valsesian record. *Alchemilla* (documented as Heimi in spring soups) and *Arctium* (recorded in soups as *chlette*) are not reported in Valsesia’s Alagna and Rimella. On the contrary, thirteen genera are documented as wild food in Alagna and Rimella in 2026, but not in Issime in 2010.

The comparison of the three Walser communities reveals similarities and divergences in phytonyms. For instance, *Carum carvi* is called Chemi in Alagna, Chrìme in Rimella, and Chummi in Issime, thus sharing the same lexeme (the common etymological root). On the contrary, *Achillea erba-rotta* subsp. *moschata* is called Ewulebi (likely originating from the French éboulis—a slope covered with loose rocks or scree) in Alagna and Gampschuchrüt (literally: chamois herb) in Rimella, despite the proximity of the two villages. These findings are broadly consistent with previous ethnobotanical work in Walser and neighboring Alpine communities, particularly in the persistence of a core group of widely used wild food taxa. At the same time, the present comparison adds a more explicit cross-community perspective by documenting differences in local plant nomenclature and in the contemporary distribution of reported uses among Issime, Alagna, and Rimella. The persistence of shared taxa alongside locally distinctive phytonyms suggests that continuity in plant knowledge does not necessarily imply uniformity across Walser communities.

These differences indicate that closely related Walser communities retain both shared and locally distinctive plant nomenclature. They may reflect interacting historical, ecological, and linguistic influences, including settlement and contact histories, local environmental conditions, and differentiation among Walser varieties. Demographic and socioeconomic changes may also affect opportunities to transmit and practice the ethnobotanical knowledge associated with these plant names. However, the present dataset does not allow these potential explanations or their relative contributions to be disentangled quantitatively. They should therefore be regarded as plausible interpretations rather than demonstrated causal relationships.

### 3.3. Medicinal Plant Knowledge

Medicinal plant knowledge was documented most extensively in Alagna (18 taxa).

The largest category involved respiratory conditions: infusions of *Thymus serpyllum* (Salegjia [A]), *Primula veris* (Peiterschlussil [A]), *Cetraria islandica* (Lickene [A]), and *Tilia platyphyllos* (Linda [A]) were used against cough, cold, and flu [32]. *Pulmonaria officinalis* (Lunkuchrud [A])—whose spotted leaves were interpreted through the doctrine of signatures as resembling the lungs—was specifically recommended for pneumonia, as the Walser name itself suggests [literally: the herb of the lungs]. The preparation rule for *Bartsia alpina*, always five or seven individuals, never more, likely encodes an implicit constraint on active compound concentrations accumulated through generational trial and error. One participant from Alagna recalled the *Artemisia* proverb transmitted from her mother: *‘Se la donna sapesse i benefici dell’artemisia, venderebbe anche la camicia’*, *‘If women knew the benefits of artemisia, they would sell even the shirt off their backs’*, encoding both pharmacological values.

**Figure 3:**
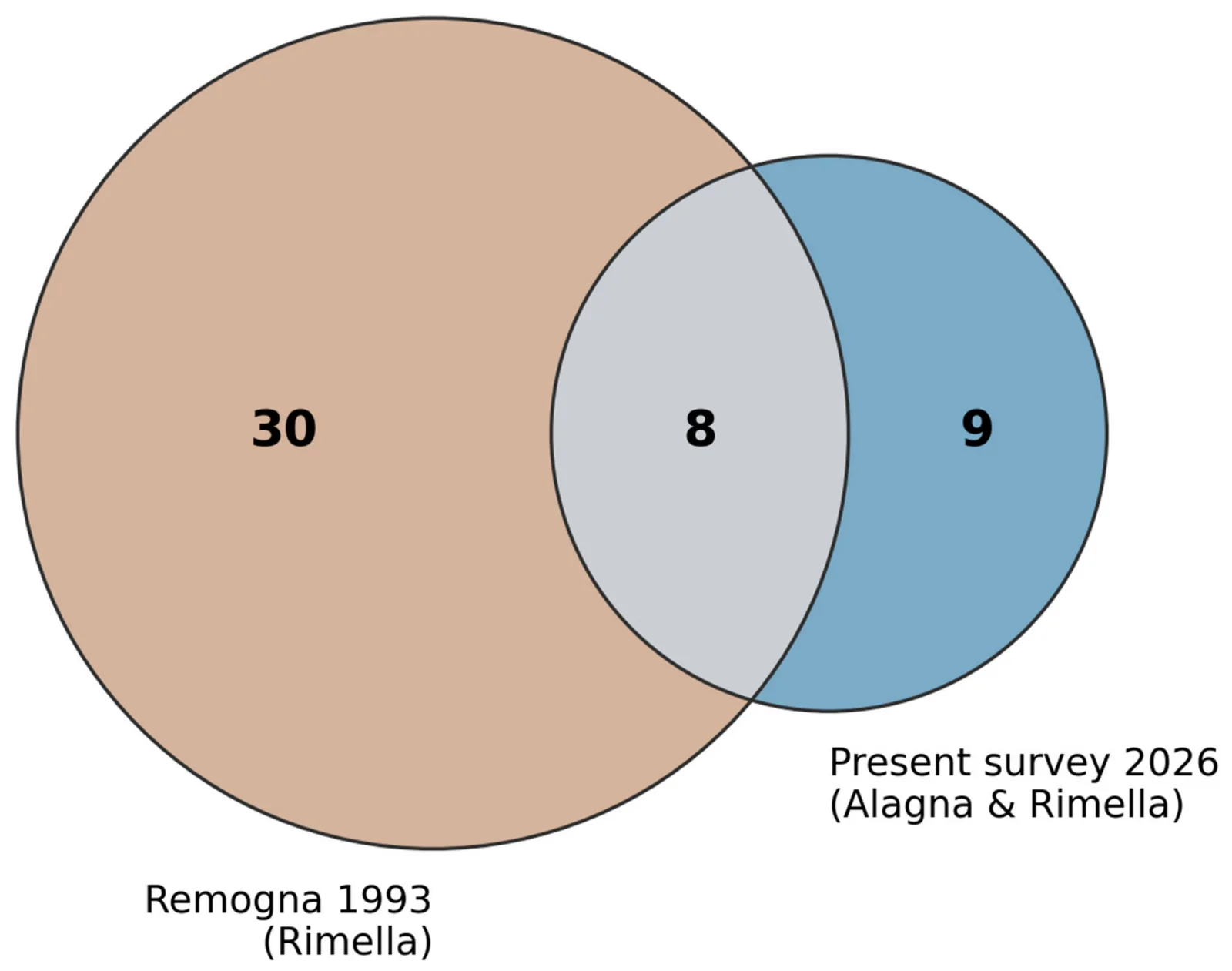
Cross-temporal Venn diagram of medicinal plant genera documented in the historical Rimella inventory of Remogna (1993; interviews with elderly Rimella informants, early 1990s [30]) and in the present Alagna and Rimella survey (2026).

The comparison of our results with Remogna [30] reveals that at genus level the two inventories share eight medicinal genera (*Arnica*, *Artemisia*, *Cetraria*, *Gentiana*, *Larix*, *Malva*, *Plantago*, and *Valeriana*), giving a Jaccard similarity of J = 0.170 (Figure 3).

This shared set includes the alpine bitters and febrifuges (*Gentiana*, *Artemisia*, the genepì group, and *Valeriana* spp.), cough remedy (*Cetraria*), the trauma and wound simples (*Arnica*, *Plantago*), the emollient *Malva*, and expectorants (*Larix*). These are precisely the taxa tied to the high pastures and the Walser therapeutic canon, and their persistence across three decades mirrors, in the medicinal register, the stable wild-food core identified in Figure 2.

Thirty medicinal genera recorded by Remogna were not recovered in the present survey. Critically, almost none are wild montane medicinal: the overwhelming majority are the cultivated and homegarden simples of the historical subsistence household—the domestic teas and condiments (*Matricaria*, *Mentha*, *Salvia*, *Rosmarinus*), the garden alliums and brassicas (*Allium*, *Brassica*), the oil, fibre and staple crops (*Linum*, *Cannabis*, *Ricinus*, *Solanum*, *Zea*, *Castanea*, *Juglans*), the imported materia medica (*Citrus*, *Myristica*), and a handful of once-common wayside and woodland remedies now fallen from use (*Verbascum*, *Polypodium*, *Asplenium*, *Ranunculus*, *Betula*, *Alnus*). Conversely, nine medicinal genera appear in the present survey but not in Remogna (*Achillea*, *Bartsia*, *Chelidonium*, *Hypericum*, *Primula*, *Pulmonaria*, *Scrophularia*, *Thymus*, *Tilia*); most are wild woodland or high-meadow medicinals whose recovery reflects the broader Alagna sampling and the structured, domain-prompted elicitation of the 2026 focus groups rather than a real expansion of the Rimellese repertoire.

### 3.4. Patterns of Continuity and Reported Change in Walser Ethnobotanical Knowledge

While we acknowledge that the presented ethnobotanical data were collected using different methods and cannot be directly compared numerically, here we present the trajectory delineated by the data collected between 2010 and 2026. The knowledge horizon has contracted simultaneously from two directions: the social (fewer people who know) and the ecological (fewer plants to know).

The most striking change was environmental: species that were still abundant in living memory (above all, *Vaccinium myrtillus*) but ecologically no longer available. Interviewees in Alagna and Rimella reported that *Vaccinium* was nearly impossible to find due to climate-driven habitat change and expanding wildlife pressure; it remains part of this core in their cultural memory, and its absence from practice signals an ecological emergency rather than knowledge loss. Nevertheless, this punctual ecological change goes along with the near-complete collapse of the local mountain food system as reported, for instance, by interviewees in Val d’Otro in 2016. One elderly male interviewee recalled that few people still actively foraged wild greens, collecting *Taraxacum* sect. *Taraxacum*, *Urtica dioica*, and *Silene vulgaris*, but that ‘the new generation did not possess the same knowledge’ or “the knowledge about the environment, about foraging, wildlife and hunting is mainly in the hands of the older generation who are worried about the little interest the younger generation expresses”. Another interviewee from Val d’Otro noted that the economic crisis of the 2010s had increased interest among young people in returning to agricultural livelihoods: ‘due to the recent financial crisis and competitive job market, more young people are currently interested in coming back’.

The changes at the social level, especially tackling the sense of community. While an interviewee declared that “the Walser had a deep love and respect for the land”, another indicated that “the sense of community is zero”, which seems to encapsulate the rupture between the relational ethic historically embedded in Walser land use and its current condition. The same informant’s phrase “*Siamo noi, non siamo io*” (“We are us, not I”) articulates a pre-market understanding of communal land tenure in which the local consortium governed pasture rights, road maintenance, and even livestock selection, the same institutional framework within which LEK, including wild-plant knowledge, was collectively managed and transmitted. The collapse of this framework, confirmed across multiple interviewees, seems to be at the base of other knowledge losses documented in this study. This process is also fostered by tourism, which the interviewees seem to associate with landscape commodification. “There is no tourism for culture now; people only come to ski or hike,” stated an informant in 2016. This was further observed by a local restaurateur in 2016 who reported that visitors consistently ordered polenta, a dish with no culinary history in the valley, illustrating the tourist-demand mechanism by which locally indifferent food becomes the public face of a place whose actual food heritage is invisible to outsiders.

## 4. Discussion

Our results evidence two main findings. First, despite ongoing knowledge erosion, there is still a stable core of wild food plants harvested among Walser communities. Second, the Walser biocultural heritage is undergoing major socioecological changes.

### 4.1. A Stable Core of Walser and Alpine Plant Knowledge

The stable core of wild food plants, represented by 14 genera (*Bistorta*, *Carum*, *Blitum*, *Fragaria*, *Knautia*, *Leontodon*, *Malva*, *Myosotis*, *Rumex*, *Silene*, *Tanacetum*, *Thymus*, *Urtica*, and *Vaccinium*), reveals their persistence across time and space: these are the genera that Walser foragers in the Italian Alps have consistently foraged for food purposes.

On the contrary, two genera that are present in the 2010 Issime dataset but absent from the 2026 Valsesian record—*Alchemilla* and *Arctium*—may have disappeared for different reasons. First, *Alchemilla* is a morphologically complex aggregate, a group of dozens of microspecies that are visually similar and require botanical expertise to distinguish, making it one of the first taxa to drop out when the chain of intergenerational transmission that taught field identification is broken. Second, the culinary use of *Alchemilla* at Issime appears already to have been peripheral in 2010: it was recorded without specific preparation context, suggesting it was known but not frequently practiced. As active knowledge-holders age and the primary teachers of plant identification disappear, genera at the periphery of practice are lost before those at the center. In contrast, *Arctium* may have disappeared due to habitat contraction. *Arctium* is a ruderal species, characteristic of pastoral disturbance zones, field margins, dung-heap peripheries, and heavily trodden paths between pastures. These habitats have contracted sharply across both the Lys Valley and the Valsesia following the decline of traditional transhumance, the downsizing of dairy herds, and the abandonment of the high-pasture rotation system [33]. As the pastoral landscape that made *Arctium* abundant and visible has contracted, so too has practical access to it, severing the experiential context in which knowledge of its edibility was maintained. The disappearance of these two genera may illustrate two distinct mechanisms of wild-food knowledge loss: attrition through the breakage of local knowledge transmission chains and attrition through the ecological erasure of the habitats that made taxa available in the first place.

Thirteen genera are documented as wild food in Alagna and Rimella 2026, but not in Issime 2010. While most of them (*Achillea*, *Carlina*, *Larix*, *Nasturtium*, *Phyteuma*, *Plantago*, *Primula*, *Sambucus*, *Sorbus*, and *Taraxacum*) were reported in a locally published book about local flora uses [34], we acknowledge that some represent genera whose primary Walser use crosses the food–medicine boundary: *Achillea erba-rotta* subsp. *moschata* (erba bianca) is used in liqueurs and rice-milk preparations as well as stomachache infusions, and *Larix decidua* shoots are eaten raw as an expectorant and altitude aid while walking. The remaining three *Cichorium*, *Oxalis*, and *Tragopogon* may not be culturally relevant in Issime, while they are certainly ecologically present, as reported in other ethnobotanical studies of the region [35].

Overall, despite these discrepancies, possibly due to methodological biases, the persistence of these core taxa suggests the existence of a culturally stable ethnobotanical kernel, composed of species that combine ecological abundance, culinary versatility, and strong mnemonic salience. Indeed, they are common to many other Alpine communities [35,36], with the exception of *Knautia arvensis* which was not previously mentioned in Alpine ethnobotanical studies, but in other Italian regions comparable “core species” patterns have been observed among Balkan mountain communities, where taxa such as *Thymus*, *Mentha*, and *Sideritis* remained resilient despite broad socio-economic change, largely because of their repeated integration into daily culinary and medicinal routines [37]. The present study extends this comparative perspective by bringing together contemporary data from three Walser communities and examining plant uses alongside local phytonyms. This makes it possible to identify both a shared ethnobotanical core and community-specific linguistic and cultural variation that may be obscured when Walser communities are considered collectively.

### 4.2. Major Changes in the Walser Biocultural Heritage: Changes in Demography, Language Attrition, Abandonment of Local Farming Practices and the Consequent Erosion of Food Memory

The most visible mechanism of biocultural heritage loss across all four study sites is demographic. Dramatic depopulation beginning in the post-World War II period, driven by industrial employment in lowland cities and by agricultural policies that systematically failed to support mountain farming, removed the community substrate in which LEK was embedded [1]. In Issime, approximately 50% of the population was employed in agriculture fifty years before our 2010 fieldwork; by the time of the fieldwork, the figure had fallen to 20%. On the one hand, the demographic erosion should be understood not as population aging, as reported in other Alpine linguistic minorities [38] and in mountain communities globally [6], and is recognized as one of the most critical structural threats to LEK survival. On the other hand, the progressive collapse of intergenerational ecological apprenticeship should also be taken into consideration. Ethnobotanical transmission depends on repeated co-presence in landscapes: walking, collecting, cooking, and discussing plants in everyday contexts. Once these routines disappear, verbal transmission alone becomes insufficient for maintaining practical competence [16,39].

Another mechanism of biocultural heritage loss is language attrition that operates as a distinct mechanism of LEK erosion, not reducible to demographic change. In 2010, at Issime, Töitschu existed only in oral form; recent efforts to establish a writing system had achieved only partial success [23]. At Rimella, the construction of a road in the second half of the twentieth century accelerated exposure to Italian-rooted dialects, initiating a linguistic substitution now far advanced. However, the interviewees confirmed that the loss of the local Walser language had already begun during fascist times, when official Italian was aggressively promoted and minority languages suppressed, which was a phenomenon common to other ethnolinguistic communities [12]. As Maffi [13] argues, the loss of a language is never simply the loss of a communication medium but the loss of a particular way of perceiving and organizing the world. The Walser botanical lexicon—with its distinct names that encode ecological observations, its functional food-use categories (lean vs. fat vegetables; segnatempo plants; plants requiring specific ritual timing), and its differential nomenclature across varieties—constitutes a cognitive infrastructure for engaging with the Alpine environment and its food resources. When that infrastructure collapses, the knowledge it supports becomes inaccessible even to community members who might otherwise be willing to receive it, because the categories in which it was encoded no longer exist in any available language. Hunn’s [14] concept of ecological indexicality captures this precisely: the Walser food-plant name points, with specificity that Italian cannot replicate, to entities and relationships in the natural world. Its loss is not a communicative inconvenience but an epistemic amputation. Recent cross-cultural ethnobotanical studies further demonstrate that linguistic erosion can take multiple trajectories. In some communities, plant nomenclature is replaced through borrowing from dominant neighboring cultures, as documented among Maronites in Cyprus, where Greek phytonyms progressively substituted Arabic ones while foraging practices partially persisted [40]. In other cases, linguistic labels survive after practical use declines, functioning as what has been called “empty shells” of former ecological engagement [18]. The Walser case appears to exhibit both processes simultaneously. The divergence across the three Walser varieties adds a further dimension. Each community has, through centuries of food practices, generated an independent ecological record embedded in its dialect variant. Homogenizing these varieties into a single ‘Walser standard’ would destroy that record.

A third mechanism is the abandonment of local farming practices. Walser botanical knowledge was embedded in a subsistence food economy built around agriculture, pastoralism, and communal management of natural resources. When this system collapsed in the post-war period, the practical context in which botanical knowledge made sense was severed. Some interviewees explicitly noted that there are fewer wild edible plants available for foraging because plant diversity has declined, as the higher pastures have been abandoned and the biodiversity-rich meadows that sustained them have retreated, as largely confirmed by the literature (e.g., [33]). The modernization of pastoralism illustrates the same dynamic: the Bruna Alpina, once kept by the Walser for her ability to climb steep mountain terrain, has been replaced by larger breeds that cannot reach the higher pastures; the communal rotation system, managed through the local consortium, has been abandoned [41]. LEK and biodiversity are co-produced and co-eroded: abandoning the food system dismantles both [5]. While our data do not allow ecological attribution in a strict quantitative sense, participants’ observations of declining bilberry availability align with broader studies showing that mountain inhabitants often detect climate-driven shifts in species abundance before such changes are fully captured in formal ecological monitoring. These local perceptions should therefore be treated as valuable indicators of environmental change rather than as definitive causal proof [17,42]. As with the wild-food core, it is the wild-gathered, high-pasture, culturally salient taxa that carry biocultural memory forward, while the plant knowledge bound to the vanished agrarian household disappears with it.

### 4.3. Food Heritage as an Anchor for LEK Revitalization

The preceding analysis points towards a conclusion with practical implications: the most effective and culturally sustainable pathway for transmitting and anchoring the broader body of Walser LEK runs through food. Food heritage, the traditional knowledge of wild plants for culinary use, their preparation, their seasonal rhythms, and their local names, is its most socially accessible and emotionally resonant dimension. The proverb about *Artemisia*, the rule against carrots in the *achmil bangada*, the distinction between lean and fat wild vegetables, and the bilberry wine that can no longer be made are not abstract ethnobotanical data points. They are food memories with strong associative power, capable of triggering intergenerational conversation in ways that more technical ecological knowledge cannot.

Documentation without transmission produces archives, not living heritage [8]. The most effective transmission is practice-based and food-centered: seasonal herb walks led by elderly knowledge-holders, cooking workshops centered on traditional wild-plant preparations, and school curricula incorporating foraging and plant identification in the local Walser variety. These activities create the contexts, the kitchen, the table, and the meadow, in which LEK has always been transmitted. The efforts in Val d’Otro to reinstall local festivities and involve all residents represent precisely this kind of revitalisation attempt; its limitation is the absence of structural support.

Local food revitalisation can serve as leverage for language revitalization. Bilingual botanical–culinary glossaries linking Walser food-plant names across all three varieties to Italian equivalents and scientific nomenclature, of which Table 1 represents a preliminary contribution, would serve simultaneously as linguistic resources, practical foraging guides, and permanent records of the divergent food histories of different communities. The FloraWalser digital database [29] represents a promising infrastructure that can be expanded through community-based documentation. The development of written forms of the three Walser varieties should maintain the distinctiveness of each and resist standardization.

Material conditions matter critically. The case of one of the interviewees, who left a career as a pastry chef to farm goats in Valsesia, milking by hand twice daily, producing cheese from June to September, and experimenting with traditional crop varieties, demonstrates that individual re-entry into traditional food practice is possible but fragile, dependent on access to land and housing. Supporting such individuals through agricultural grants, rural housing schemes, and land access programmes is therefore a concrete policy intervention with direct implications for the pace of LEK loss. WWOOFing programmes, through which volunteers learn traditional food skills in exchange for labour, represent an additional mechanism for knowledge exchange that partially compensates for the reduced capacity for intergenerational transmission, although the mobility of these volunteering schemes may not favour the persistence of such knowledge.

Finally, LEK revitalization through food heritage requires active ecosystem management. Several Alagna participants reported that wild bilberries (*Vaccinium myrtillus*) have become nearly impossible to find; elderberry (*Sambucus nigra*) jam production has been disrupted by expanding magpie populations attributed to broader ecological change. Where the plants that culinary knowledge is about are no longer available in the forms that knowledge assumes, cultural revitalization alone is insufficient. Restoration of traditional grazing regimes that maintain biodiversity-rich mountain meadows is a necessary complement to any food-heritage revitalization programme. Future ethnobotanical research may need to move beyond urgent documentation alone and toward intervention-oriented models that actively rebuild ecological practice. Recent theoretical work argues that the next frontier of ethnobiology lies in fostering communing platforms, educational infrastructures, and community-led ecological engagement capable of restoring plant–human relationships rather than merely archiving their decline [43].

### 4.4. Methodological Considerations and Limitations

Several methodological considerations should be taken into account when interpreting the comparisons presented here. First, the ethnobotanical datasets were collected in different years, communities, and research contexts, using different interview and recording procedures. The 2010 Issime study involved informal interviews, plant walks, free-listing, and participant observation among elderly women, whereas the 2026 Alagna and Rimella studies employed semi-structured interviews and focus-group or individual interview formats. The 2016 Val d’Otro research had a different objective and was primarily concerned with socioeconomic change rather than comprehensive plant documentation. These differences mean that the datasets cannot be interpreted as equivalent repeated measurements of the same population.

Second, participants were primarily older knowledge holders. This was appropriate for documenting retained ethnobotanical knowledge, but it does not allow us to quantify the distribution of knowledge across generations or directly measure the magnitude of community-level LEK loss. The absence of a plant or practice from the contemporary dataset may reflect changes in knowledge, use, ecological availability, sampling intensity, or differences in elicitation. Conversely, the presence of a plant in both historical and contemporary records demonstrates continuity in the available records but does not establish uninterrupted transmission across generations.

Third, the comparison with historical ethnobotanical sources should therefore be interpreted as a qualitative diachronic assessment. More systematic evaluation of LEK erosion would require repeated sampling using comparable protocols, inclusion of different age cohorts, and explicit assessment of intergenerational knowledge transmission. The present study can nevertheless provide a baseline for such future research by documenting currently retained plant knowledge, local phytonyms, and participants’ accounts of social, linguistic, and ecological change.

The absence of a plant or practice from a contemporary dataset cannot by itself be interpreted as evidence of knowledge loss, since it may also reflect differences in ecological availability, local practices, sampling intensity, or elicitation.

## 5. Conclusions

In Issime in 2010, elderly women fell silent when asked about wild herbs, then began to speak, memories returning to childhood mornings collecting plants with their mothers and to flavours no longer found in any kitchen. In Val d’Otro in 2016, D.E., one of the last people who still actively foraged wild greens described the knowledge passing away with his generation. In Alagna in 2026, participants recalled that bilberries are now nearly impossible to find and that the magpies have taken the elderberries. In Rimella in 2026, an elderly woman remembered that the whitebeam and the wild spinach share the name *malpene*, a linguistic fossil of famine-food practices now centuries old.

These are facets of a single process, documented here across sixteen years: the progressive rarefaction of a biocultural heritage, 43 documented plant taxa (culinary and medicinal), three differentiated Walser dialect varieties, a rich body of culinary and pharmacological knowledge, seasonal rituals, and ecological observations that has sustained human communities in one of Europe’s most demanding environments for eight centuries. The comparison of historical and contemporary records documents substantial continuity in parts of the Walser ethnobotanical repertoire alongside reported changes in plant use, language, demographic context, and opportunities for intergenerational knowledge transmission. Because the datasets were collected using different methods and from different communities, these findings should not be interpreted as a quantitative measure of the rate or magnitude of LEK erosion. Rather, they identify patterns that are consistent with processes of ethnobotanical transformation and increasing vulnerability of knowledge transmission. The Walser language and its varieties are not merely the medium through which botanical knowledge is transmitted; they are part of its content, encoding ecological observations and food histories that cannot be recovered once the linguistic infrastructure that carried them is gone.

We argue that food heritage, the practical revitalization of traditional wild-plant food cultures, offers the most effective anchor for this broader revitalization effort. Food practices are the most socially accessible, emotionally immediate, and intergenerationally transmissible dimension of Walser’s biocultural heritage. The central question is therefore no longer only how much knowledge remains, but under what ecological and social conditions knowledge can continue to be practiced, renewed, and made meaningful for future generations. Cooking workshops, communal foraging, and school-based food education within the local Walser variety provide contexts in which LEK can be transmitted and renewed. Heritage that is not practiced is not preserved: it is only mourned. Future research should complement ethnobotanical documentation with systematic intergenerational comparisons, standardized elicitation protocols, and repeated surveys of the same communities. Such approaches would allow researchers to distinguish more clearly between changes in plant availability, changes in use, and changes in the distribution and transmission of ethnobotanical knowledge.

## Figures and Tables

**Table 1:** Description of study sites and datasets.

Site	Year	Study Component	Participants	Methods and Data Collection
Issime, Aosta Valley	2010	Ethnobotanical inventory	15 elderly women	Informal interviews, plant walks, free-listing and participant observation.
Alagna, Valsesia	2026	Ethnobotanical inventory	6 participants, aged 69–88	Semi-structured focus group; audio-recorded with consent.
Rimella, Valsesia	2026	Ethnobotanical inventory	16 individual interviewees plus approximately 10 additional brief interviews	Interviews facilitated by local museum curator; audio-recorded with consent.
Val d’Otro, Valsesia	2016	Supplementary contextual ethnography	8 interviewees	Two multi-day field visits and semi-structured interviews; Italian and partly English; audio-recorded with consent.

**Table 2:** Foraged plant taxa recorded across the Walser communities of Issime (I), Alagna (A), and Rimella (R), with their original Walser dialect names.

Botanical Taxon and Family	Walser Local Names (Dialect Variety)	Parts Used	Recorded Local Uses
*Achillea erba-rotta* subsp. *moschata* (Wulfen) Vacc./Asteraceae	Ewulebi (A); Gampschuchrüt (R)	Flowers	F: Liqueurs, rice-milk (riso-latte); digestive; collected exclusively above 2000 m a.s.l. M: Stomachache infusions; digestive bitters
*Alchemilla vulgaris* aggr. L./Rosaceae	Heimi (I)	Leaves	F: Spring soups (minestre verdi)
*Arctium lappa* L./Asteraceae/UNISGSANG15004	Chlette (I)	Leaves	F: Soups
*Aria edulis* (Willd.) M.Roem. /Rosaceae	Malbaum (A); Malpumbem (R)	Fruits	F: Berries eaten especially by children
*Arnica montana* L./Asteraceae/UNISGSANG18006	Schneiberger (A); Ŝchantunbljömu (R)	Flowers	M: Ointment for trauma and bruises; external use only
*Artemisia genipi* Weber ex Stechm./Asteraceae/UNISGSANG15006	Schenupi [male] (A)	Flowers	M: Digestive genepì liqueur; calming infusion; menstrual pain relief
*Artemisia umbelliformis* Lam./Asteraceae	Schenupi [female, more bitter] (A)	Flowers	M: Same uses as *A. genipi*; considered more bitter and more potent
*Bartsia alpina* L./Orobanchaceae	—	Aerial parts	M: Fever decoction; must use odd number of plants (5 or 7), never more—implicit dosage regulation
*Bistorta officinalis* Delarbre/Polygonaceae	Zunga (A); Gràsch (R); Parrictu (I)	Leaves	F: Spring soups; consistently mentioned across all sites
*Carlina acaulis* L./Asteraceae	Driesching (A); Schtecchju (R)	Receptacle	F: Shepherds’ snack; flowers open in sun and close before rain—natural barometer (segnatempo)
*Blitum bonus-henricus* (L.) Rchb./Amaranthaceae	Haimini (A); Malpene (R); Lucche (I)	Leaves	F: Omelettes, risottos, soups; classified as ‘fat vegetable’. Leaves applied to relieve nettle stings (A)
*Carum carvi* L./Apiaceae/UNISGSANG15010	Chemi (A); Chrìme (R); Chummi (I)	Seeds	F: Seasoning; children ate seeds as sweets in Carcoforo and Rimella
*Cetraria islandica* (L.) Ach./Parmeliaceae/UNISGSANG15011	Lichkene (A); Liken (R)	Whole thallus	M: Decoction for cough
*Chelidonium majus* L./Papaveraceae	—	Latex	M: Applied to warts; external use only (toxic internally)
*Cichorium intybus* L./Asteraceae	Schwinubliäma (R)	Roots	F: Roasted as a coffee substitute
*Fragaria vesca* L./Rosaceae/UNISGSANG18047	Eckbérru (I)	Fruits, leaves	F: Preserves, soups
*Gentiana lutea* L./Gentianaceae/UNISGSANG18027	Janzuwirza (A); Janzenu (R)	Roots	M: Digestive in grappa; rubbed on children’s nails to prevent nail-biting
*Hypericum perforatum* L./Hypericaceae/UNISGSANG15021	—	Flowering aerial parts	M: Oil for burns and wound healing; macerated in olive oil for 40 days from 24 June
*Knautia arvensis* (L.) Coult./Caprifoliaceae	Veisthénnji (I)	Leaves	F: Soups
*Larix decidua* Mill./Pinaceae	Leirch (A); Lìrch (R)	Young shoots, wood	M: Raw young shoots as expectorant at altitude; weather gauge (segnatempo)
*Leontodon hispidus* L./Asteraceae	Leituji (I)	Leaves	F: Soups, omelettes
*Malva sylvestris* L./Malvaceae/UNISGSANG15025	Molva (A); Màlvu (R); Moalvi (I)	Flowers	M: Decoctions for stomachache; tisanes (I)
*Myosotis sylvatica* Ehrh. ex Hoffm./Boraginaceae/UNISGSANG18011	Lljibigottschaugjini (I)	Leaves	F: Pan-fried with cheese; soups
*Nasturtium officinale* W.T.Aiton/Brassicaceae/UNISGSANG18012	Chressu (A); Chrasschu (R)	Leaves	F: Salads; gathered near water springs (R)
*Oxalis acetosella* L./Oxalidaceae/UNISSANG15027	Surala (A)	Leaves, stems	F: Salads; acidic flavour
*Phyteuma spicatum* L./Campanulaceae	Zunge (A)	Leaves	F: Soups; turns black when cooked; ‘lean vegetable’ requiring fat accompaniment
*Plantago lanceolata/P. major* L./Plantaginaceae/UNISGSANG15031	Beneditschja (A)	Leaves	F: Soups. M: Leaves used on wounds with morning dew (Rimella)
*Primula veris* L./Primulaceae	Peiterschlussil (A); Fikke (R)	Flowers, leaves	F: Leaves in soups or salads (R). M: Infusions for cough (A)
*Pulmonaria officinalis* L./Boraginaceae	Lunkuchrud (A)	Leaves, flowers	M: Expectorant; pneumonia and bronchitis l
*Rumex acetosa* L./Polygonaceae/UNISGSANG15038	Souru/Kottulljunhghji (I)	Leaves	F: Soups, omelettes
*Rumex alpinus* L./Polygonaceae/UNISGSANG15039	Blacka (A); Blàkchtu (R); Blackhu (I)	Leaves, stems	F: Capunet (stuffed leaf rolls); boiled stems; also livestock fodder. Present across all three communities
*Sambucus nigra* L./Adoxaceae/UNISSANG15040	Holderna (A); Holdernu (R)	Fruits	F: Jam from ripe berries
*Scrophularia nodosa* L./Scrophulariaceae	Nachtschazt (A)	Leaves	M: Leaves applied reversed to disinfect wounds
*Silene dioica* (L.) Clairv./Caryophyllaceae	Hoasunblétt (I)	Leaves	F: Soups
*Silene vulgaris* (Moench) Garcke/Caryophyllaceae/UNISGSANG15044	Spïchal (A); Schpeckene (R); Kwicki (I)	Leaves	F: Omelettes, risottos, soups; ‘fat vegetable’, sufficient alone. Pan-Alpine Walser staple
*Tanacetum balsamita* L./Asteraceae	Peiterschrud (A)	Leaves	F: Soups, omelettes
*Taraxacum officinale* aggr. F.H.Wigg./Asteraceae/UNISSANG15048	Grosa (A); Sàletu der màtte (R)	Leaves	F: Raw salads; pan-cooked; sometimes confused with *Cichorium intybus*
*Thymus serpyllum* aggr. L./Lamiaceae/UNISGSANG15049	Salegjia (A); Béérchallje (R); Chollje (I)	Leaves, flowers	F: Spice in ricotta ravioli filling. M: Infusions for cough and flu. Present across all sites
*Tilia platyphyllos* Scop./Malvaceae	Linda (A); Ljendu (R)	Flowers	M: Infusions for cough
*Tragopogon pratensis* L./Asteraceae/UNISGSANG18051	Habermorch (A); Védru (R)	Young shoots	F: Cooked as an asparagus substitute (R)
*Urtica dioica* L./Urticaceae/UNISGSANG18055	Nessia (A); Nassju (R); Néssju (I)	Leaves	F: Omelettes, risottos, ravioli; fibres for textiles. Core species across all communities
*Vaccinium myrtillus* L./Ericaceae/UNISGSANG15057	Haita/Blowsberri (A); Hepter (R); Heitu (I)	Fruits, leaves	F: Bilberry wine; preserved in grappa (I)
*Valeriana celtica* L./Caprifoliaceae	Bockuchrud (A); Sàjunkà (R)	Roots	M: Fever, pneumonia, bronchitis; roots placed in stored clothes to repel moths

A = Alagna (Titzschu variety of Walser); R = Rimella (Tittschu variety of Walser); I = Issime (Éischemtöitschu variety of Walser); F: food use; M: herbal/medicinal use.

## Data Availability

The original contributions presented in this study are included in the article. Further inquiries can be directed to the corresponding authors.
